# Measuring Time-of-Flight in an Ultrasonic LPS System Using Generalized Cross-Correlation

**DOI:** 10.3390/s111110326

**Published:** 2011-10-31

**Authors:** José Manuel Villladangos, Jesús Ureña, Juan Jesús García, Manuel Mazo, Álvaro Hernández, Ana Jiménez, Daniel Ruíz, Carlos De Marziani

**Affiliations:** 1 Department of Electronics, Polytechnic School, University of Alcalá, Ctra. Madrid-Barcelona Km. 33,600, Alcalá de Henares, Madrid 28805, Spain; E-Mails: villa@depeca.uah.es (J.M.V.); jesus.urena@uah.es (J.U); mazo@depeca.uah.es (M.M.); alvaro@depeca.uah.es (A.H.); ajimenez@depeca.uah.es (A.J.); daniel.ruiz@depeca.uah.es (D.R.); 2 Department of Electronic Engineering, National University of Patagonia San Juan Bosco, Ciudad Universitaria, Ruta Provincial n° 1 s/n, km. 4, Comodoro Rivadavia (Chubut) 9000, Argentina; E-Mail: marziani@unpata.edu.ar (C.D.M.)

**Keywords:** generalized cross-correlation, ultrasonic LPS, phase transform, Kasami codes

## Abstract

In this article, a time-of-flight detection technique in the frequency domain is described for an ultrasonic Local Positioning System (LPS) based on encoded beacons. Beacon transmissions have been synchronized and become simultaneous by means of the DS-CDMA (Direct-Sequence Code Division Multiple Access) technique. Every beacon has been associated to a 255-bit Kasami code. The detection of signal arrival instant at the receiver, from which the distance to each beacon can be obtained, is based on the application of the Generalized Cross-Correlation (GCC), by using the cross-spectral density between the received signal and the sequence to be detected. Prior filtering to enhance the frequency components around the carrier frequency (40 kHz) has improved estimations when obtaining the correlation function maximum, which implies an improvement in distance measurement precision. Positioning has been achieved by using hyperbolic trilateration, based on the Time Differences of Arrival (TDOA) between a reference beacon and the others.

## 1. Introduction

The term LPS (Local Positioning System) refers to a system used for location and positioning in indoor (or at least reduced) environments where the use of positioning techniques based on GPS is very limited due to the weakness of the GPS signals. These kinds of systems are the keystone for the implementation of intelligent spaces able to interact with people and robots. Nowadays there are a lot of applications that can be developed in modern buildings with an effective LPS [1]. As examples the guidance of people inside buildings [2], the study of the people’s behavior in some contexts [3], the development of healthcare and entertainment applications [4] or some indoor robot applications [5] can be highlighted.

LPSs have been developed using different technologies such as infrared [6], RF [7], or ultrasound [8], when ultrasonic signals are used there are several beacons emitting in the covered area and different mobile devices whose positions are going to be calculated. In this process, the system measures the time of arrival (TOA) or the time-differences of arrival (TDOA) to estimate the distance or distance difference between each mobile node and the different beacons. TDOA permits that the emitters and each receiver to not be synchronized by means of radio-frequency or infrared signals (asynchronous detection), which simplifies the hardware; the disadvantage is that it is necessary to use one more beacon as reference. Due to the existence of several emitters sharing the same channel, it is essential to use a method to avoid the interferences between them, like frequency division for multiple access (FDMA), time division for multiple access (TDMA) or code division for multiple access (CDMA). The last one is a spread spectrum technique that consists of codifying each emission with a different code, and then allowing the identification of each beacon code at the receiver among all the received signals. CDMA is well known and widely used in ultrasonic applications [9].

The determination of TOA (or TDOA) implies the measurement of time delay in the arrival of a signal (or difference of delays in the arrival of two signals) to a receiver. In the case of natural sounds, including speech, intense work has been developed during the last years. The methods used deal with the random nature of these signals and their high relative bandwidth. Different algorithms have been compared in [10] in terms of the suitable sampling frequency to implement them, in the time domain (standard cross correlation -CC-, generalized cross correlation -GCC-) and in the frequency domain and envelope analysis (generalized phase spectrum -GPS-, envelope combined with GPS). In the case analyzed in this paper, the signals used are in the ultrasonic frequencies band for air with a lower relative bandwidth than sound. They are also coded and modulated before they are transmitted. Additionally different signals emitted from different beacons share simultaneously the same channel and are received with only one receiver.

**Figure 1.**
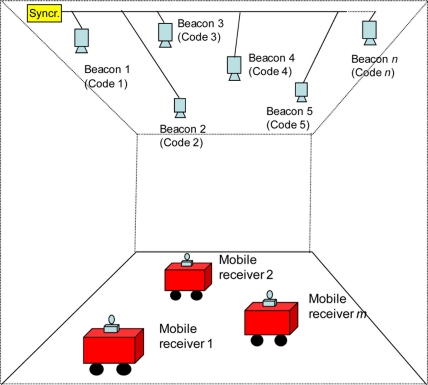
Block diagram of the ultrasonic LPS.

This paper presents an ultrasonic LPS, such as that shown in Figure 1, based on a group of transmitter beacons located at known positions, which transmit simultaneously and periodically by using DS-CDMA (Direct-Sequence Code Division Multiple Access) technique [11]. Each beacon emits a BPSK-modulated 255-bit Kasami code with a 40 kHz carrier [12]. The modulation symbol is formed by one or several carrier cycles (*m*) in order to provide more energy to the channel. An important feature of this LPS is the absence of synchronization between the beacons and the receiver. This means that it becomes necessary to use a positioning algorithm based on hyperbolic trilateration, by using as distance measurement the time differences of arrival (TDOA) between a reference beacon and the others, given the asynchronous feature of the LPS described. In order to estimate every signal’s instant of arrival at the receiver, the received signal is sampled and stored in a temporary buffer with enough sample size to store at least one complete transmission period (approximately 40 ms).

**Figure 2.**
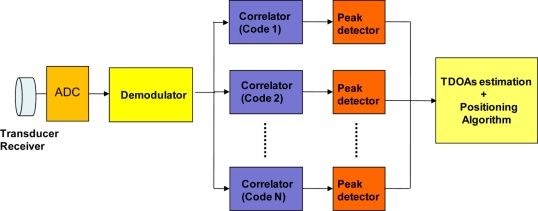
Block diagram of the receiver.

As has been mentioned, each beacon has assigned a code or sequence for encoding the emission. The arrival instant for each emission is often determined by the correlation between the received signal and the code to be detected. This process is known as *matched filtering*, and it is based on detecting at what instant it provides a correlation maximum. A block diagram of the receiver is shown in Figure 2, which basically consists of an acquisition system based on an 8-bit ADC (fs = 400 kHz) and a high-speed processing unit based on a DSP or FPGA [13].

The Inter-Symbol Interference (ISI) caused by the fact that the Auto-Correlation (AC) function of the sequences used, and the Cross-Correlation (CC) between them, is not null near the maximum, implies that it is sometimes difficult to detect the real position of the maxima [14]. In order to improve the detection process, without applying typical communications algorithms as MAI (Multiple Access Interference) or ISI cancellation, which usually involve a high computational load, the use of generalized cross-correlation (GCC) is proposed [15], which is widely employed in acoustics to estimate the delay time between signals [16,17].

This article is organized as follows: in Section 2 the positioning system model reported here is described, and the classical detection process based on cross-correlation. In Section 3, GCC is explained, as well as its application to detect sequences or codes assigned to each beacon. In Section 4, some results obtained for the detection process by applying GCC are provided, and compared with those obtained from CC. Finally, some conclusions are discussed in Section 5.

## 2. Positioning System Model

**Figure 3.**
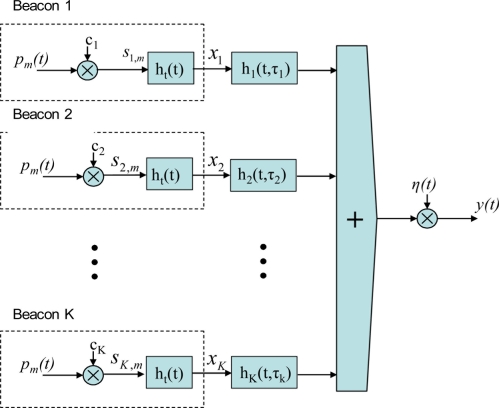
Block diagram of the proposed positioning system model.

In the positioning system model (see Figure 3) the beacons are considered as a set of *K* transmitters. Every emitted signal, *x_j_(t)*, is the encoded emission of the beacon *j* and is the result of the BPSK modulation of a binary code *c_j_* with a carrier *p_m_(t)*, and the convolution of this modulated signal with the impulse response of the transmitter, *h_t_(t)*. Considering that *h_j_(t*, *τ_j_)* is the response of the channel for the beacon *j*, *τ_j_* is the delay in receiving the sequence assigned to beacon *j* and *η(t)* is a zero-mean Gaussian noise and variance *σ^2^*, the received signal *y*(*t*) is given by Equation (1):

(1) $$y(t)=\sum_{j=1}^{K}x_{j}(t)*h(t,\tau_{j})+\eta (t)$$

and in terms of discrete-time, the received signal *y*[*n*] is defined by Equation (2):

(2) $$y[n]=\sum_{j=1}^{K}x_{j}[n]*h_{j}[n]+\eta [n]$$

By applying the convolution process, the received signal *y*[*n*] becomes:

(3) $$y[n]=\sum_{j=1}^{K}\sum_{l=0}^{P}h_{j}[l]\cdot x_{j}[n-l]+\eta [n]$$

where *x_j_*[*n*] is the discrete-time version of the transmitted signal by the beacon *j*; *K* is the number of beacons; *P* is the number of samples in the analysis window or capture buffer; *h_j_*[*l*] is the impulse response of the physical channel between the beacon *j* and the receiver, characterised by signal delay and attenuation; and *η*[*n*] is a zero-mean Gaussian noise.

Considering the detection process of a beacon *j*, it is necessary to carry out the correlation between the received signal *y*[*n*] and the sequence (or code) to be detected *s_j_*[*n*]. The correlation function is given by Equation (4):

(4) $$\varphi_{s_{j}}[n]=\sum_{k=-\infty }^{\infty }y[k]s_{j}[n+k]=\alpha_{j}R_{s_{j},s_{j}}[n-D_{j}]+\sum_{\underset{i\neq j}{i=1}}^{K}R_{y,s_{i}}[n]+R_{\eta ,s_{j}}[n]$$

Both the auto-correlation function of the sequence transmitted by the beacon *j* (when *x_i_* = *x_j_*) and the cross-correlations between the sequence transmitted by the beacon *j* and the other beacons can be identified in Equation (4). The last term in Equation (4) represents the cross-correlation between the sequence to be detected and the noise. The function is maximum for *n = D_j_* which corresponds to the arrival instant of the sequence assigned to the beacon *j*. The second term in Equation (4) is the cross-correlation between all the other sequences *i* (*i* ≠ *j*) or beacons and the received signal *y*[*n*], and represents the interference due to multiple access (MAI). The last term means the correlation between noise and the sequence assigned to the beacon *j*. The arrival instant of the sequence *j*, in samples, is provided by Equation (5):

(5) $$D_{j}=\text{arg}\;\underset{n}{\text{max}}\varphi_{s_{j}}[n]$$

The time difference of arrival (TDOA) between a reference beacon *i* and another beacon *j*, considering the sampling time *Ts*, is given by Equation (6):

(6) $$\begin{array}{l} \tau_{\mathrm{ij}}=Ts\cdot D_{\mathrm{ij}}\\ =\mathrm{Ts}\left(\text{arg}\;\underset{n}{\text{max}}\varphi_{s_{i}}[n]-\text{arg}\;\underset{n}{\text{max}}\varphi_{s_{j}}[n]\right) \end{array}$$

## 3. Generalized Cross-Correlation

Another method for computing the arrival instant of a sequence *j* assigned to a beacon is based on the inverse Fourier transform of the cross-spectral density between the received signal and the sequence to be detected. This method is known as Generalized Cross-Correlation (GCC), and it is widely used by many authors as a method for estimating the delay in reception of a signal at two receivers positioned at a specific distance from a communication source [15]. In particular, it is commonly used for the detection of an acoustic signal source and for locating it in a determined environment. In this case, the receivers are a set of microphones [18].

If it is considered that the received signal *y*(*t*) and the signal to be detected *x*(*t*), have been filtered prior to carrying out the cross-correlation process by using the filters *H_y_*(*f*) and *H_x_*(*f*), respectively, the cross-spectral density, related to the cross-correlation, is defined by Equation (7):

(7) $${\tilde{G}}_{\mathrm{xy}}^{\mathrm{GCC}}(f)=H_{x}(f)H_{y}^{*}(f)G_{\mathrm{xy}}(f)$$

where *H_y_*(*f*) and *H_x_*(*f*) are the frequency response of the filters of the received signal *y*(*t*) and the signal to be detected *x*(*t*), and *G_xy_*(*f*) is the cross-spectral density between them. The generalised cross-correlation function is obtained from its inverse transform in Equation (8):

(8) $$R_{\mathrm{xy}}^{\mathrm{GCC}}(\tau )=F^{-1}\left\{\Phi (f)G_{\mathrm{xy}}(f)\right\}$$

where Φ(*f*) = *H_x_*(*f*)H_y_*(*f*) represents the correlation filter.

Regarding a discrete-time analysis, the generalised cross-correlation in discrete time, between the received signal *y*[*n*] and the sequence *s_j_*[*n*] to be detected, is given by Equation (9):

(9) $$\varphi_{s_{j}}^{\mathrm{GCC}}[n]=\sum_{k=0}^{N-1}\Phi_{j}[k]S_{j}[k]Y^{*}[k]e^{\mathrm{ik}\frac{2\pi }{N}n}$$

where Y*[*k*] is the conjugate of the discrete Fourier transform for the discrete version of the received signal *y*[*n*]; *S_j_*[*k*] and Φ*_j_*[*k*] are the discrete Fourier transforms of the sequence *s_j_*[*n*] to be detected and the weight function respectively. The weight function represents a previous filtering of the received signal and the sequence to be detected, which accentuates the peak or the maximum of the cross-correlation function at the instant of arrival of the sequence. In the frequency domain, this filter is equivalent to applying a weight function to the cross-spectral density function between the received signal and the sequence to be detected. As E{*S_j_*[*k*]Y*[*k*]} = *G_S_j_,y_*[*k*] represents the cross-spectral density between *y*[*n*] and *s_j_*[*n*], where the operators E{·} and * are the expected value and the conjugated complex operator; then, Equation (9) can be expressed as Equation (10):

(10) $$\varphi_{s_{j}}^{\mathrm{GCC}}[n]=\sum_{k=0}^{N-1}\Phi_{j}[k]G_{s_{j},y}[k]e^{\mathrm{ik}\frac{2\pi }{N}n}$$

Note that if Φ*_j_*[*k*] = 1, the GCC function Equation (10) becomes the standard version of the cross-correlation of the two signals obtained from the inverse Fourier transform for their cross-spectral density. An approximation to the expression gives an estimation of the GCC function by considering a limited number *N* of samples. The estimated instant *n = D_j_* corresponds to the maximum of the function in Equation (10), and represents the arrival of the sequence *j* at the receiver, which is given by Equation (11):

(11) $${\hat{D}}_{j}=\text{arg}\;\underset{n}{\text{max}}\varphi_{s_{j}}^{\mathrm{GCC}}[n]$$

Filtering has two objectives. First, it accentuates the signal to be correlated in those frequencies where the signal-to-noise ratio is higher; therefore, *Φ_j_(f)* depends on the signal spectrum and noise. Secondly, the filtering yields the most accentuated peak possible at the correct instant for obtaining a good temporary resolution. However, the accentuated maxima are more sensitive to errors produced by finite observation times, particularly in the case of low signal-to-noise ratios. The choice of the filter is thus a trade-off between temporary resolution and stability. The way in which this calculation is carried out has been studied by several authors, taking into account two criteria: temporary resolution and error due to noise. Several filtering expressions have been analysed in [15].

The filtering function known as PHAse Transform (PHAT filtering), is widely used to estimate signal delay between two receivers located at a given distance from the acoustic source or transmitter. Its expression in this case, considering beacons as transmitters whose signal is *s_j_*[*n*], and the received signal *y*[*n*], is provided in Equation (12):

(12) $$\Phi_{j}[k]=\frac{1}{\left|S_{j}[k]Y[k]\right|}$$

The advantage over the other filtering functions analysed in [15] is that it notably improves estimation in environments with a certain level of reverberation. Assuming that the noise is completely uncorrelated, peaks in the correlation function are much narrower, near a true delta function at the instant of arrival of the sequence, with an absence of sidelobes. This is because the delay information is present in certain frequency phases and it is not affected by the transform, since the applied filtering enhances the real instant of arrival of the sequence or delay, and eliminates spurious delays, to a greater or lesser extent, depending on noise power.

### Detection of the Signal from the Beacons

**Figure 4.**
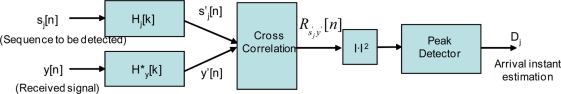
Block diagram of the estimation process of the arrival instant for a sequence *j* based on GCC.

Since beacons are considered as emitters whose transmitted signal is *s_j_*[*n*], and the received signal *y*[*n*], the estimation of the arrival instant for each beacon, when applying the GCC with PHAT filtering, requires a more complex analysis in order to apply the correlation between the received signal and the sequence to be detected, as the received signal is a composition of as many emitting sources or signals as available beacons. Figure 4 shows the estimation process of the arrival instant of sequences for GCC with a pre-filtering function, in time domain.

Let consider the received signal from the beacons, defined as Equation (13):

(13) $$y[n]=\sum_{j=1}^{K}s_{j}[n]*h_{j}[n]+\eta [n]$$

where *K* is the number of beacons; *S_j_*[*n*] is the sequence assigned to the beacon *j*; *h_j_*[*n*] is the channel response for the beacon *j*; and *η*[*n*] is the noise. The cross power spectrum between the received signal *y*[*n*] and the sequence *S_j_*[*n*] to be detected is Equation (14):

(14) $$\begin{array}{l} \Phi_{y,s_{j}}(\omega )=Y(\omega )X_{j}^{*}(\omega )=\left(\sum_{j=1}^{K}S_{j}(\omega )H_{j}(\omega )+N(\omega )\right)S_{j}^{*}(\omega )\\ \;\;\;\;\;\;\;\;\;\;\;\;\;\;\;\;\;=S_{1}(\omega )S_{j}^{*}(\omega )e^{-j\omega D_{1}}+S_{2}(\omega )S_{j}^{*}(\omega )e^{-j\omega D_{2}}+\ldots \\ \;\;\;\;\;\;\;\;\;\;\;\;\;\;\;\;\;+S_{j}(\omega )S_{j}^{*}(\omega )e^{-j\omega D_{j}}+\ldots +S_{K}(\omega )S_{j}^{*}(\omega )e^{-j\omega D_{K}}+N(\omega )S_{j}^{*}(\omega )\\ \;\;\;\;\;\;\;\;\;\;\;\;\;\;\;\;\;=\Phi_{s_{1},s_{j}}(\omega )e^{-j\omega D_{1}}+\Phi_{s_{1},s_{j}}(\omega )e^{-j\omega D_{2}}+\ldots +\Phi_{s_{j},s_{j}}(\omega )e^{-j\omega D_{j}}+\ldots +\Phi_{s_{K},s_{j}}(\omega )e^{-j\omega D_{K}}+N(\omega )S_{j}^{*}(\omega ) \end{array}$$

where Φ*_s_i_,s_j__* (*ω*) is the cross spectrum between each transmitted sequence *S_i_*[*n*] and the sequence *S_j_*[*n*] to be detected. The cross correlation between the received signal and the sequence *j*, based on the inverse Fourier transform, Equation (15):

(15) $$\begin{array}{lll} R_{y,s_{j}}[k] & = & F^{-1}\left(\Phi_{y,s_{j}}(\omega )\right)\\ & = & \sum_{\underset{i\neq j}{i=1}}^{K}R_{s_{i},s_{j}}[n]*\delta [n-D_{i}]+R_{s_{j},s_{j}}[n]*\delta [n-D_{j}]+R_{N,s_{j}}[n]\\ & = & \sum_{\underset{i\neq j}{i=1}}^{K}R_{s_{i},s_{j}}[n-D_{i}]+R_{s_{j},s_{j}}[n-D_{j}] \end{array}$$

where *R_s_i_,s_j__* [*n* − *D_j_*] is the auto-correlation of the sequence *S_j_*[*n*] to be detected, and *R_s_i_,s_j__* [*n* – *D_i_*] is the correlation between each transmitted sequence *S_i_*[*n*] (apart from that to be detected), and the sequence to be detected *S_j_*[*n*]. It is assumed that *R_N,s_j__* [*n*] = 0 since noise is considered uncorrelated. By applying the phase transform (PHAT), the weight function or filter becomes Equation (16):

(16) $$\Psi_{j}(\omega )=\frac{1}{\left|{\hat{\Phi }}_{y,s_{j}}(\omega )\right|}$$

where Φ̂*_y,s_j__* (*ω*) is the estimation of the cross power spectrum between the received signal and the sequence to be detected, computed from the estimation of their spectra *Ŷ* (*ω*) and *Ŝ_j_* (*ω*), respectively. The GCC estimated with the PHAT filter is defined by Equation (17):

(17) $$\begin{array}{l} {\hat{R}}_{y,s_{j}}^{\left(\mathrm{gcc}\right)}[k]=\frac{1}{2\pi }\int_{-\pi }^{\pi }\frac{1}{\left|{\hat{\Phi }}_{y,s_{j}}(\omega )\right|}\cdot {\hat{\Phi }}_{y,s_{j}}(\omega )\cdot e^{j\omega k}d\omega \\ =\frac{1}{2\pi }\int_{-\pi }^{\pi }\left(\frac{1}{\left|\sum_{\underset{i\neq j}{i=1}}^{K}{\hat{\Phi }}_{s_{i},s_{j}}(\omega )\cdot e^{-j\omega D_{i}}+{\hat{\Phi }}_{s_{j},s_{j}}(\omega )\cdot e^{-j\omega D_{j}}\right|}\right)\cdot \left(\sum_{\underset{i\neq j}{i=1}}^{K}{\hat{\Phi }}_{s_{i},s_{j}}(\omega )\cdot e^{-j\omega D_{i}}+{\hat{\Phi }}_{s_{j},s_{j}}(\omega )\cdot e^{-j\omega D_{j}}\right)\cdot e^{j\omega k}d\omega \end{array}$$

In this case, it is possible to state Equation (18):

(18) $$\left|\sum_{\underset{i\neq j}{i=1}}^{K}{\hat{\Phi }}_{s_{i},s_{j}}(\omega )\cdot e^{-j\omega D_{i}}+{\hat{\Phi }}_{s_{j},s_{j}}(\omega )\cdot e^{-j\omega D_{j}}\right|\neq \sum_{\underset{i\neq j}{i=1}}^{K}{\hat{\Phi }}_{s_{i},s_{j}}(\omega )\cdot e^{-j\omega D_{i}}+{\hat{\Phi }}_{s_{j},s_{j}}(\omega )\cdot e^{-j\omega D_{j}}$$

Thus, ${\hat{R}}_{y,s_{j}}^{\left(\mathrm{gcc}\right)}\;[k]$ is not an ideal delta, but an attenuated delta at instant *D_j_* and some sidelobes. This is provided by the other sequences that interfere in the correlation process due to their pseudo-orthogonality. Furthermore, the more the signal-to-noise ratio reduces, the more the detection process worsens due to the spectral contribution of the noise power. An alternative is to use, as a PHAT filter, the inverse module of the Fourier transform of the auto-correlation of the sequence to be detected. This is:

(19) $$\begin{array}{l} {\hat{R}}_{y,s_{i}}^{\left(\mathrm{gcc}\right)}[k]=\frac{1}{2\pi }\int_{-\pi }^{\pi }\frac{1}{\left|{\hat{\Phi }}_{s_{j},s_{j}}(\omega )\right|}\cdot {\hat{\Phi }}_{y,s_{j}}(\omega )\cdot e^{j\omega k}d\omega \\ =\frac{1}{2\pi }\int_{-\pi }^{\pi }\left(\frac{1}{\left|{\hat{\Phi }}_{s_{j},s_{j}}(\omega )\right|}\right)\cdot \left(\sum_{\underset{i\neq j}{i=1}}^{K}{\hat{\Phi }}_{s_{i},s_{j}}(\omega )\cdot e^{-j\omega D_{i}}+{\hat{\Phi }}_{s_{j},s_{j}}(\omega )\cdot e^{-j\omega D_{j}}\right)\cdot e^{j\omega k}d\omega \\ =\frac{1}{2\pi }\int_{-\pi }^{\pi }\left(\frac{1}{\left|{\hat{\Phi }}_{s_{j},s_{j}}(\omega )\right|}\right)\cdot \left(\sum_{\underset{i\neq j}{i=1}}^{K}{\hat{\Phi }}_{s_{i},s_{j}}(\omega )\cdot e^{-j\omega D_{i}}\right)\cdot e^{j\omega k}d\omega \\ +\frac{1}{2\pi }\int_{-\pi }^{\pi }\left(\frac{1}{\left|{\hat{\Phi }}_{s_{j},s_{j}}(\omega )\right|}\right)\cdot \left({\hat{\Phi }}_{s_{i},s_{j}}(\omega )\cdot e^{-j\omega D_{i}}\right)\cdot e^{j\omega k}d\omega \end{array}$$

As the auto-correlation spectrum is a real and positive function:

(20) $$\left|{\hat{\Phi }}_{s_{j},s_{j}}(\omega )\right|={\hat{\Phi }}_{s_{j},s_{j}}(\omega )=S_{j}\cdot S_{j}^{*}$$

It becomes:

(21) $$\begin{array}{l} {\hat{R}}_{y,s_{i}}^{\left(\mathrm{gcc}\right)}[k]=\frac{1}{2\pi }\int_{-\pi }^{\pi }(\frac{1}{\left|{\hat{\Phi }}_{s_{j},s_{j}}(\omega )\right|})\cdot \left(\sum_{\underset{i\neq j}{i=1}}^{K}{\hat{\Phi }}_{s_{i},s_{j}}(\omega )\cdot e^{-j\omega D_{i}}\right)\cdot e^{j\omega k}d\omega \\ +\frac{1}{2\pi }\int_{-\pi }^{\pi }\left(e^{-j\omega D_{j}}\right)\cdot e^{j\omega k}d\omega \end{array}$$

As can be observed, the searched sequence becomes a unitary delta, whereas the deltas for the other sequences are weighted by Equation (22). Since the sequences are pseudo-orthogonal, their values become much less than one.

(22) $$\frac{{\hat{\Phi }}_{s_{i},s_{j}}(\omega )}{{\hat{\Phi }}_{s_{j},s_{j}}(\omega )}\;\mathrm{with}\;\;i\neq j$$

## 4. Results

### 4.1. Simulation Results

**Figure 5.**
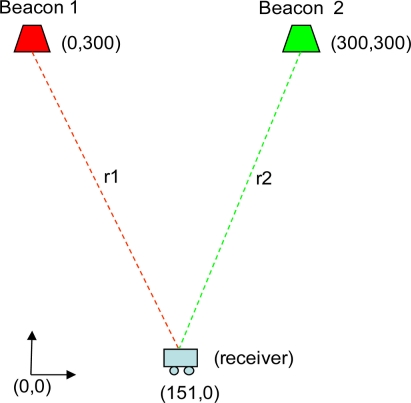
Position of the beacons and the receiver for the analysis of the detection process.

In order to compare the sequence detection method based on cross-correlation (CC) between the received signal and the sequence assigned to each beacon with the method based on generalised cross-correlation (GCC) and on applying phase transform (PHAT) filtering, a LPS with two beacons have been considered. The beacons have been located in a 2D system at positions (*x_1_* = 0, *y_1_* = 300 cm) and (*x_2_* = 300 cm, *y_2_* = 300 cm), whereas the receiver is located at (*r_x_* = 0, *r_y_* = 151 cm). In other words, the receiver is practically equidistant from both beacons (see Figure 5). For the emission, 255-bit *Kasami* codes, with one carrier cycle as modulation symbol (*m* = 1), have been used.

**Figure 6.**
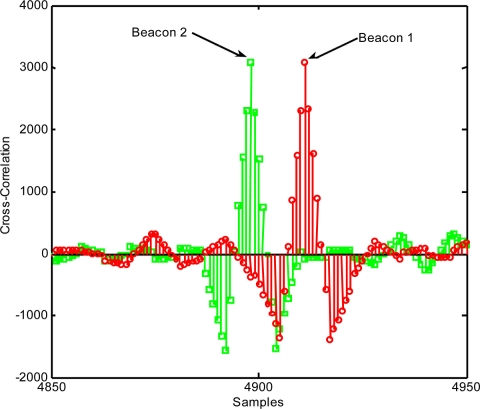
Beacon detection in the absence of noise by applying the cross-correlation (CC) method in the time domain.

Figure 6 shows the detection process in the absence of noise and considering ideal beacons, based on cross-correlation between the received signal and the sequences to be detected in the time domain. It can be observed the correlation function maxima, as well as the appearance of sidelobes as a consequence of the ultrasonic signal modulation process, which generates a widening of the spectrum.

**Figure 7.**
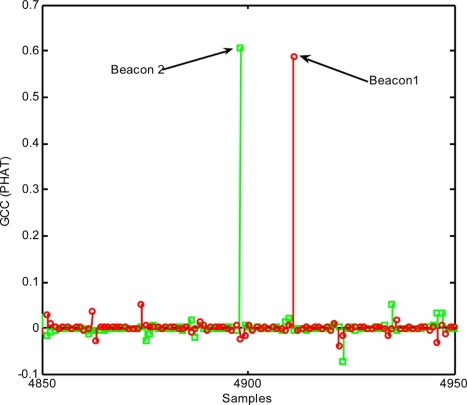
Beacon detection in the absence of noise, by applying generalised cross-correlation (GCC) with PHAT filtering.

Figure 7 shows how the method based on GCC with PHAT filtering noticeably improves detection: sidelobes have practically disappeared, and the detection instant becomes almost a delta.

**Figure 8.**
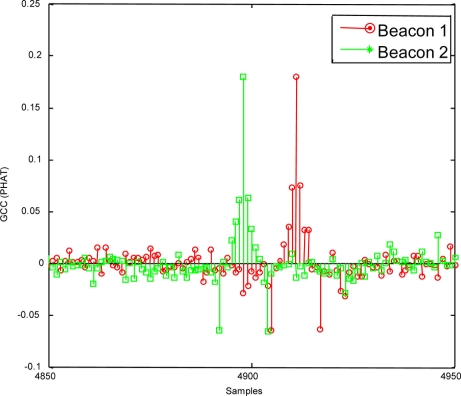
Beacon detection with a signal-noise ratio of 0 dB, by applying generalised cross-correlation (GCC) with PHAT filtering.

Prior filtering of the signals based on phase transform (PHAT) accentuates the frequencies around the carrier. Then, when the Fourier transform-based correlation is applied, a delta is obtained at the sequence arrival instant. Figure 8 shows the detection process for a signal-to-noise ratio of 0 dB, where the GCC method still improves detection, despite the appearance of sidelobes in the frequency response generated by noise, which implies high values around the carrier frequency.

**Figure 9.**
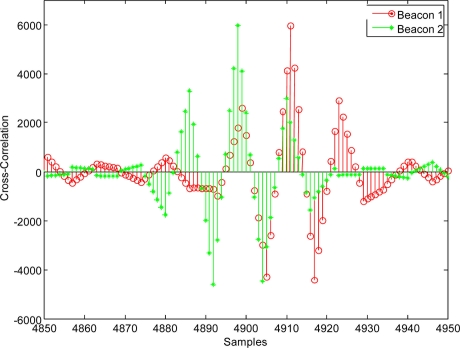
Beacon detection by applying CC, for a modulation symbol formed by four carrier cycles (*m* = 4).

**Figure 10.**
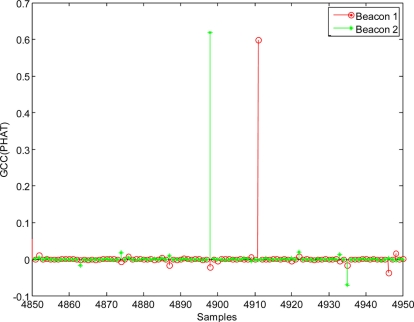
Beacon detection by applying generalised cross-correlation (GCC) with PHAT filtering, for a modulation symbol formed by four carrier cycles (*m* = 4).

In the case of a modulation symbol consisting of four carrier cycles (*m* = 4), to increase the energy transmitted by the beacons and concentrate the transmission spectrum around the carrier frequency, the edge effects caused by modulation when CC is applied are shown in Figure 9, whereas the detection corresponding to sequence arrival instant when GCC is applied is illustrated in Figure 10.

### 4.2. Real Results

**Figure 11.**
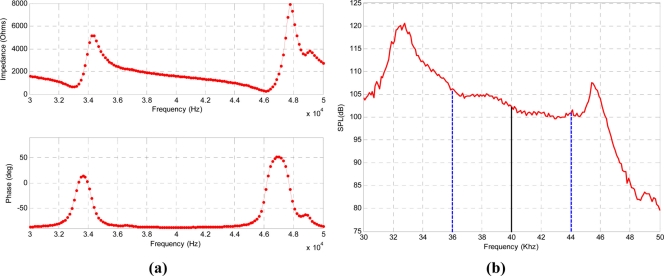
Measured 328ST160 transducer performance: **(a)** Impedance/Phase angle *vs.* frequency; **(b)** Sound pressure level (SPL) *vs.* frequency.

In order to analyse the GCC behaviour with real signals a practical set-up with a geometric configuration similar to that used for simulations has been arranged and tested. A Prowave (328ST160) transducer [19] has been selected to implement the beacons. The measured impedance/phase angle *versus* frequency for this transducer can be seen in Figure 11(a) and the sound pressure level (SPL) *versus* frequency in Figure 11(b). The transducer has been used working around the frequency of 40 kHz in order to have enough bandwidth (8 kHz) and to minimize the phase distortion in the emitted signal.

**Figure 12.**
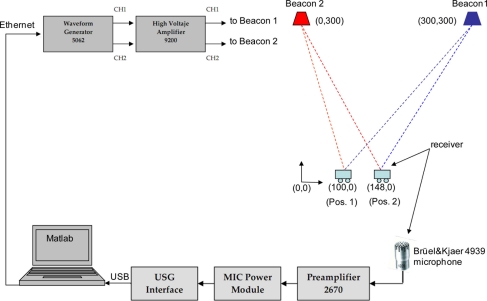
Experimental setup diagram.

Two beacons have been used for transmission and the receiver has been positioned on the floor. Figure 12 shows the experimental setup diagram. The beacons are excited by a dual-channel Tabor 5062 Arbitrary Waveform Generator followed by a Tabor 9200 voltage amplifier. The receiver is a Brüel & Kjaer 4939 microphone with a 2670 preamplifier which output has been captured by an UltraSoundGate 116 Hm, an Avisoft ultrasonic signal acquisition system [20]. A computer enables the acquisition at 400 kHz sampling frequency and performs all the post processing using Matlab.

**Figure 13.**
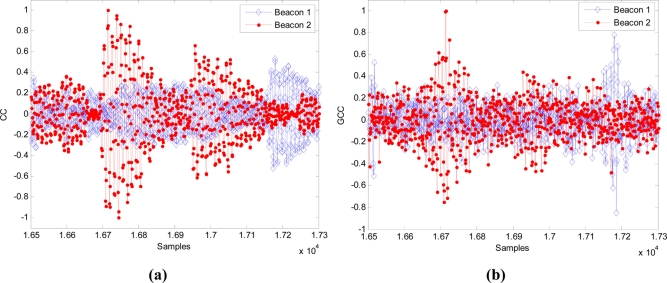
Beacon detection in the position 1 with real signals (*m* = 1) applying: **(a)** CC and **(b)** GCC.

When the receiver is in the position 1 (according to the diagram shown in Figure 12), the detection process results for a modulation symbol with one carrier cycle (*m* = 1) by applying CC and GCC, are shown in Figure 13(a,b), respectively.

**Figure 14.**
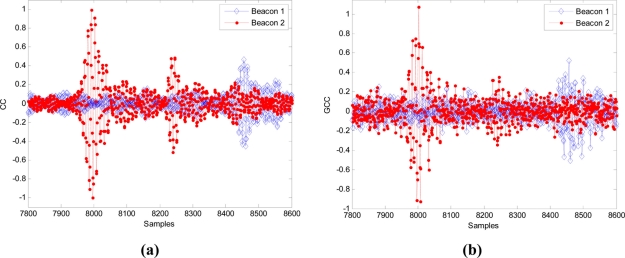
Beacon detection in the position 1 with real signals (*m* = 4) applying: **(a)** CC and **(b)** GCC.

In the same position 1, the results obtained when using a modulation symbol with four carrier cycles (*m* = 4) by applying CC and GCC, are shown in Figure 14(a,b), respectively.

In both cases (with *m* = 1 in Figure 13 and with *m* = 4 in Figure 14), it can be observed that the use of the GCC provides a significant enhancement of the peaks regarding the lateral values affected by the modulation process and the characteristics of the transmission channel.

**Figure 15.**
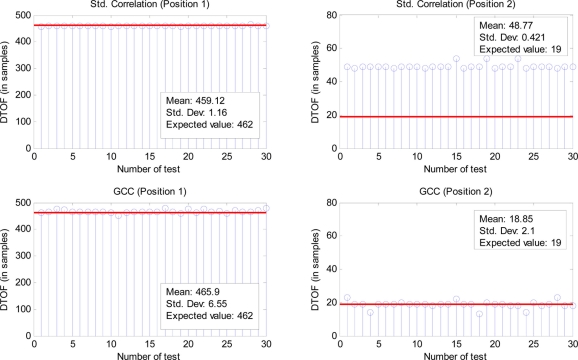
DTOAs measured in positions 1 and 2 when emit beacons 1 and 2 (Figure 12) using standard correlation (CC) and GCC.

Figure 15 shows the results obtained, using standard correlation (CC) and GCC, in the measurement of the DTOA between the signals emitted from the beacons 1 and 2 when they are received in positions 1 and 2 (according to Figure 12). The results are expressed in samples (the sampling period used is 2.5 μs), as the difference between the maximum peaks of both correlations, the corresponding to beacons 1 and 2. The test has been repeated 20 times, and the values of the mean and the standard deviation have been included for every case. The expected values for position 1 and 2 have been measured by using a laser distance meter.

**Figure 16.**
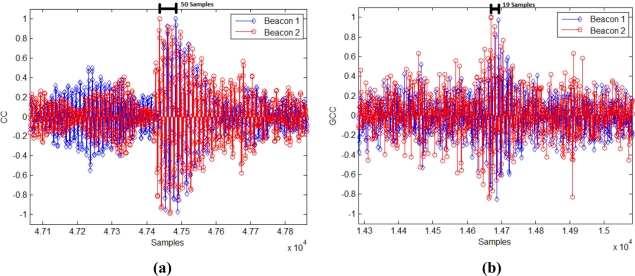
Beacon detection in the position 2 with real signals (*m* = 1) applying: **(a)** CC and **(b)** GCC.

For the position 1, in which both signals arrive with a considerable delay, CC gives a most repetitive result (although looking at Figures 13 and 14, the main peak in the correlations is clearer for GCC). Nevertheless, for the position 2, in which both signals arrive almost simultaneously, the interference between them affects more to the CC, giving an erroneous result in the computation of the DTOA. In this case the GCC allows computing the DTOA correctly. Figure 16(a,b) show the output of the correlation process for the signals received in this position 2 by using CC and GCC respectively. It can be verified how the use of GCC gives better results because the main peaks in every representation have less secondary peaks around.

## 5. Conclusions

A method based on the GCC for obtaining the TDOA of two signals coming from different beacons to the same receiver has been proposed in this work. It has been applied to an ultrasonic LPS, whose beacons emit encoded signals by means of Kasami sequences. The detection algorithm is based on the cross-spectral density between the received signal and the modulated sequences to be detected (each one related to its corresponding beacon).

The proposed algorithm provides a significant reduction in the lateral lobe effects, when compared with standard correlation, caused by the modulation process, the interference between emissions and the characteristics (attenuation, multipath, *etc*.) of the channel. The detection of the maximum peak to determine the arrival instant of each sequence is then easier.

The algorithm has been tested with simulated and real data comparing its performance with that obtained when a basic correlation (CC) is used. GCC gives always a clearer maximum peak than CC with less significant secondary peaks around the main one. When the signals arrive to the receiver with a considerable delay (milliseconds) the performance of both detection algorithms, CC and GCC, is similar, being the CC less sensible to the noise and distortion introduced by the transducer. On the other hand, when the signals arrive to the receiver almost simultaneously (very low DTOA), the GCC allows to compute the DTOA while the CC fails (because the interference between both signals and its influence in the secondary peaks).
